# CD38 Predicts Favorable Prognosis by Enhancing Immune Infiltration and Antitumor Immunity in the Epithelial Ovarian Cancer Microenvironment

**DOI:** 10.3389/fgene.2020.00369

**Published:** 2020-04-30

**Authors:** Ying Zhu, Zhigang Zhang, Zhou Jiang, Yang Liu, Jianwei Zhou

**Affiliations:** ^1^Department of Gynecology, The Second Affiliated Hospital, Zhejiang University School of Medicine, Zhejiang University, Hangzhou, China; ^2^Key Laboratory of Tumor Microenvironment and Immune Therapy of Zhejiang Province, Hangzhou, China

**Keywords:** CD38, ovarian cancer, prognosis, tumor-infiltrating lymphocytes, antitumor immunity

## Abstract

The identification of predictive biomarkers and novel targets to optimize immunotherapy strategies for epithelial ovarian cancer (EOC) is urgently needed. CD38 is a multifunctional glycoprotein that acts as an ectoenzyme and immune receptor. However, the underlying immunological mechanisms and prognostic value of CD38 in EOC remain unclear. CD38 gene expression in EOC was evaluated by using Gene Expression Profiling Interactive Analysis (GEPIA) and TISIDB database. The prognostic value was calculated using GEPIA and Kaplan–Meier plotter. Gene set enrichment analysis was conducted to study the roles of CD38 in the EOC microenvironment. Furthermore, the relationship between CD38 expression level and immune cell infiltration was analyzed by the Tumor Immune Estimation Resource and TISIDB. The GEPIA and TISIDB databases showed that CD38 expression in EOC was higher than that in normal tissue and was highest in the immunoreactive subtype among the four molecular types. A total of 424 cases from GEPIA revealed that high levels of CD38 were associated with longer disease-free survival [hazard ratio (HR) = 0.66, *P* = 0.00089] and increased overall survival rate (*HR* = 0.67, *P* = 0.0016). Kaplan–Meier plotter also confirmed the prognostic value of CD38 in EOC. Data from The Cancer Genome Atlas database demonstrated that gene signatures in many categories, such as immune response and adaptive immune response, were enriched in EOC samples with high CD38 expression. In addition, CD38 was positively correlated with immune cell infiltration, especially infiltration of activated CD8^+^ T cells, CD4^+^ T cells, and B cells. CD38 is positively correlated with prognosis and immune cell infiltration in the EOC microenvironment and contributes to the regulation of antitumor immunity. CD38 could be used as a prognostic biomarker and potential immunotherapy target.

## Introduction

Epithelial ovarian cancer (EOC) is the seventh most common cancer and seriously threatens female health worldwide (Siegel et al., 2019). There are no typical early symptoms and feasible screening options, and the majority of ovarian cancer patients present with late or advanced disease (stages III and IV) (Bowtell et al., 2015; Menon et al., 2018). The standard curative treatments involve cytoreductive surgery followed by platinum-based chemotherapy. Despite improvements in therapy, relapse is inevitable, and the 5-year overall survival (OS) for EOC is approximately only 45% (Lheureux et al., 2019b). Currently, multitarget immunotherapy has become one of the most promising approaches in cancer therapy. In particular, immune checkpoint blockade, with targets such as PD-1, PD-L1, and CTLA-4, has emerged as a novel therapeutic method with noteworthy results in malignant melanoma and lung cancer (Ribas and Wolchok, 2018; Scott et al., 2018). In general, immunotherapy is less efficient in patients with EOC and lacks biomarkers for selecting the optimal population for immunotherapy (Odunsi, 2017; Lheureux et al., 2019a). Therefore, coping with the challenges and exploiting more effective immunotherapeutic approaches depend on a better understanding of the tumor–immune interactions in the tumor microenvironment (TME) (Mandal and Chan, 2016).

CD38 is a 45-kDa type II transmembrane glycoprotein with ectoenzymatic functions, defined as an ectoenzyme, which participates in the catabolism of nicotinamide adenine dinucleotide (NAD^+^) to ADP-ribose and cyclic ADP-ribose (Niels et al., 2018; Hogan et al., 2019), thus playing an important role in adenosinergic pathways and mediating NAD^+^ homeostasis. In addition, CD38 has also been described as a surface differentiation marker for lymphocytes, including plasma cells, myeloid cells, and other lymphoid cells (Hogan et al., 2019; Joosse et al., 2019). Because CD38 is uniformly and highly expressed on myeloma cells, a novel therapeutic strategy has emerged that involves targeting CD38 in multiple myeloma; basic research and clinical trials have demonstrated that anti-CD38 mAbs (such as daratumumab) have high efficacy and favorable safety as immunotherapies to increase survival for multiple myeloma patients (Dimopoulos et al., 2016; Horenstein et al., 2019). Recently, studies have also demonstrated that CD38 is involved in CD8^+^ T-cell suppression via adenosine receptor signaling in the TME, which can cause resistance to PD-1/PD-L1 blockade therapy (Chen et al., 2018). These results showed that CD38 plays multifaceted functional roles in lymphocytes and in the TME. However, the underlying immunological mechanisms and prognostic value of CD38 in the microenvironment of EOC are still unclear.

Here, we used online databases, such as Gene Expression Profiling Interactive Analysis (GEPIA), Oncomine, TISIDB, and Kaplan–Meier plotter (Supplementary Table S1), to validate that CD38 was highly expressed in EOC compared with normal ovarian tissue and positively correlated with good prognosis. CD38 was correlated with tumor-infiltrating lymphocytes (TILs), especially with activated CD8^+^ T cells. These findings uncover the important immunoregulatory role of CD38 in the EOC microenvironment and provide a potential target for ovarian cancer immunotherapy.

## Materials and Methods

### GEPIA Database Analysis

Gene Expression Profiling Interactive Analysis^1^ is a comprehensive web-based analysis tool that includes tumor and normal sample RNA sequencing data from The Cancer Genome Atlas (TCGA) and Genotype-Tissue Expression projects and provides analysis of the interactive relationship, functions, and prognostic value of gene expression in cancer and normal tissues (Tang et al., 2017). The mRNA expression level and prognostic predictive significance of the CD38 gene in EOC were determined in GEPIA. Moreover, gene expression correlation analysis was also conducted by using the GEPIA database.

### Oncomine Database Analysis

Oncomine^2^ is a gene chip–based online database (Rhodes et al., 2004) that was employed to further verify the expression level of CD38 in EOC.

### TISIDB Database Analysis

TISIDB^3^ is an integrated repository web portal for analysis of interactions between tumors and the immune system (Ru et al., 2019). It integrates multiple types of data resources in oncoimmunology, including literature mining results from the PubMed database and TCGA. The TISIDB was used to assess the role of CD38 in tumor–immune interplay.

### Kaplan–Meier Plotter Database Analysis

Kaplan–Meier plotter^4^ is an online database integrating gene expression data and clinical information (Gyorffy et al., 2012). To evaluate the prognostic value of CD38 mRNA expression in ovarian cancer, CD38 was entered into this database to obtain Kaplan–Meier survival plots. The hazard ratio (HR) with 95% confidence intervals and log-rank *P* values were calculated on the web page.

### The Tumor Immune Estimation Resource Database Analysis

The Tumor Immune Estimation Resource (TIMER)^5^ is a user-friendly web interface for investigating the molecular characterization of tumor–immune interactions (Li et al., 2017). TIMER adopts a deconvolution of previously published computational approaches for estimating the abundance of TILs from gene expression profiles. Approximately six subsets of TILs were pre-calculated in 32 cancer types and data from the TCGA database. The correlations between CD38 mRNA expression and gene markers of TILs were analyzed via correlation modules in TIMER.

### TCGA Data Downloading

The level 3 gene expression profile for EOC using Affymetrix HT Human Genome U133a (version September 8, 2017) was downloaded from TCGA datasets^6^. Meanwhile, clinicopathological and survival information were also obtained from the TCGA data portal. The ESTIMATE algorithm (Estimation of STromal and Immune cells in MAlignant Tumor tissues using Expression data) was used to calculate immune scores and stromal scores of ovarian cancer by applying the downloaded data. The ESTIMATE algorithm was designed by Yoshihara et al. This algorithm can analyze specific gene expression signatures of immune and stromal cells to calculate immune and stromal scores (Yoshihara et al., 2013) and finally predict the non-tumor cell infiltration level.

### Gene Set Enrichment Analysis

Gene set enrichment analysis (GSEA) was performed to identify significantly enriched groups of genes (Subramanian et al., 2005). In this study, GSEA software^7^ was applied to analyze biological pathway divergences between high and low CD38 mRNA in the EOC expression profiles of TCGA data. *P* < 0.05 and FDR (false discovery rate) *q* < 0.05 were considered threshold values to estimate statistical significance.

### Calculation of Immune and Stromal Scores

The Cancer Genome Atlas level 3 gene expression data and clinical information were acquired from the Genomic Data Commons (GDC, available at https://portal.gdc.cancer.gov/) data portal on May 10, 2019. Immune and stromal scores were calculated by the ESTIMATE algorithm of the downloaded data for each ovarian cancer sample (Yoshihara et al., 2013). The cutoff values were defined with median scores, and based on the cutoff value, samples were divided into low and high immune/stromal score groups. The survival analysis was assessed by the log-rank test. *P* < 0.05 was considered statistically significant.

### Statistical Analysis

Survival analysis of CD38 in EOC was performed by using Kaplan–Meier plotter and GEPIA, and these two databases used the log-rank test for hypothesis evaluation. The Cox proportional hazard ratio and the 95% confidence interval are displayed in the survival curves. The thresholds for high-/low-expression-level cohorts were defined as the median CD38 mRNA level. The correlation of CD38 mRNA expression was assessed by using TIMER and TISIDB. Spearman correlation was calculated, and *P* < 0.05 indicated statistically significant differences.

## Results

### Expression Levels of CD38 mRNA in EOC

**FIGURE 1:**
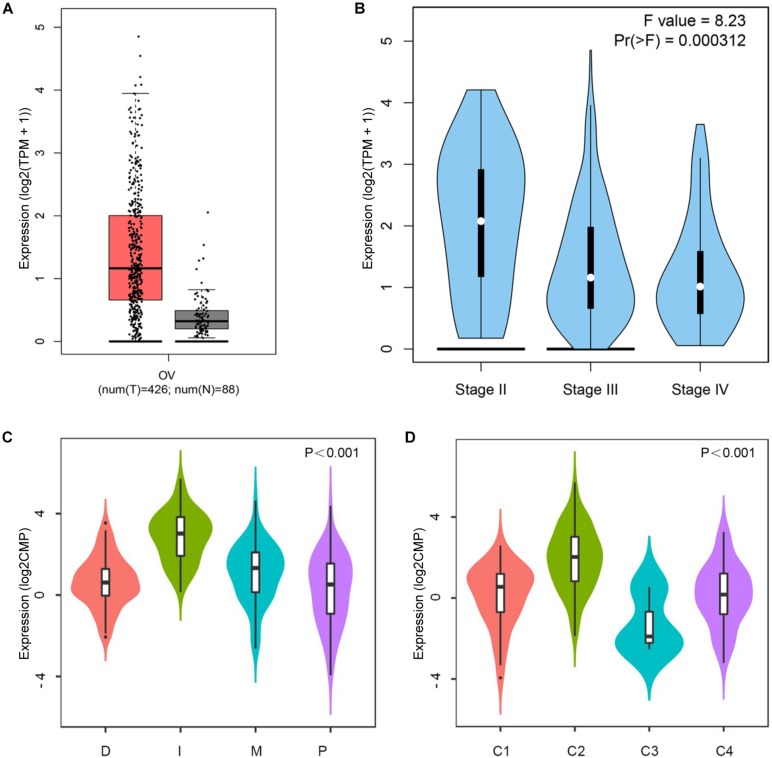
CD38 expression levels in ovarian cancer. **(A)** Epithelial ovarian cancer compared with normal tissues in the GEPIA database. **(B)** CD38 expression levels in different stages of epithelial ovarian cancer from GEPIA database. **(C)** CD38 expression levels in different molecular subtypes of epithelial ovarian cancer from TISIDB database. **(D)** CD38 expression levels in different immune subtypes of epithelial ovarian cancer from TISIDB database. The CD38 gene expression profiles were normalized by log2(TPM + 1) in **(A,B)**, and log counts per million mapped reads (log2CPM) in **(C,D)**.

Based on the data of the GEPIA database, the CD38 mRNA levels in EOC and normal ovarian tissues were assessed. The results showed that the CD38 expression level in EOC was higher than that in normal ovarian tissue (Figure 1A). In addition, when compared to the different stages of EOC in some data sets, higher expression was observed in stage II, and lower expression was observed in stages III and IV (Figure 1B). Unfortunately, data about stage I disease were not found. We further used the Oncomine database to examine CD38 expression in multiple histological types of EOC. This analysis revealed that CD38 mRNA was more highly expressed in malignant EOC than in borderline tumors, and ovarian endometrioid carcinoma had lower CD38 expression than ovarian serous cancer (Supplementary Figure S1).

Four molecular subtypes (mesenchymal, immunoreactive, differentiated, and proliferative) have been identified in EOC (Konecny et al., 2014). In TISIDB, we found that CD38 expression was highest in the immunoreactive subtype and lowest in the proliferative subtype (Figure 1C). This result implied that CD38 was strongly linked to the tumor immune microenvironment. Shmulevich’s study clustered six immune subtypes for cancer (Thorsson et al., 2018). In TISIDB, we further analyzed CD38 expression in different immune subtypes of EOC. We found CD38 was expressed in four types, including C1 (wound healing type), C2 [interferon γ (IFN-γ) dominant type], C3 (inflammatory type), and C4 (lymphocyte depleted type). CD38 was highest in the C2 (IFN-γ dominant) type and lowest in the C3 (inflammatory) type (Figure 1D).

### The Prognostic Value of CD38 in EOC

**FIGURE 2:**
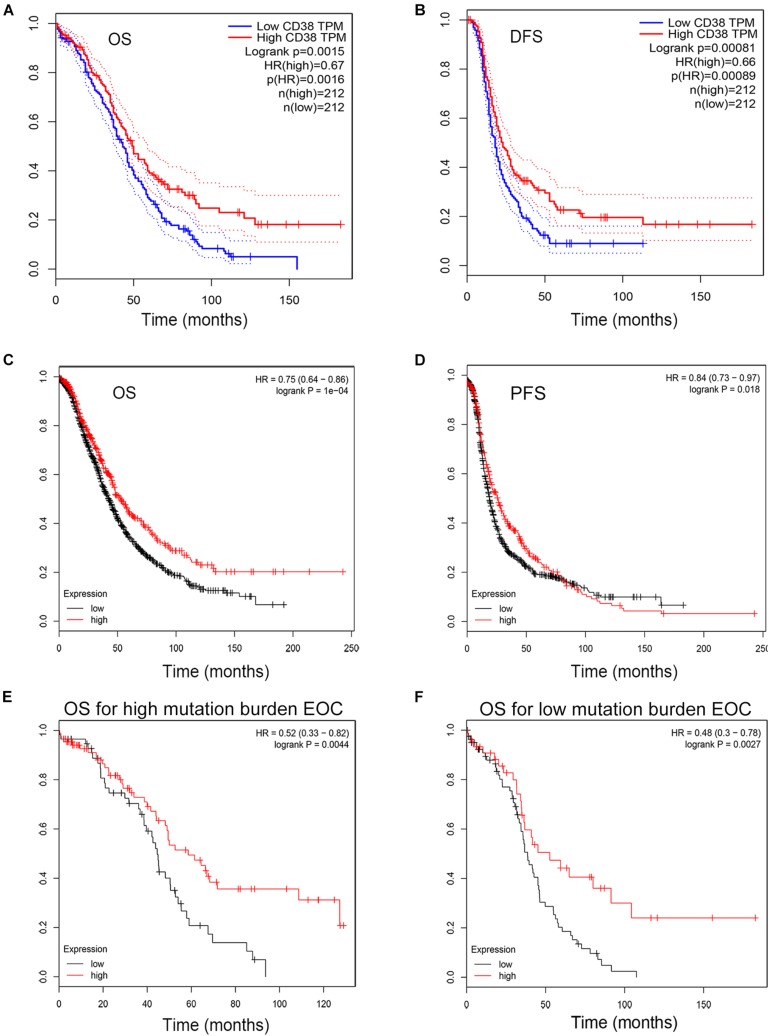
Kaplan–Meier survival curves comparing the high and low expression of CD38 in epithelial ovarian cancer in the GEPIA and Kaplan–Meier plotter databases. **(A,B)** Survival curves of OS and DFS in ovarian cancer from GEPIA databases. **(C,D)** Survival curves of OS and PFS in epithelial ovarian cancer from Kaplan–Meier plotter databases. **(E,F)** High CD38 expression was correlated with better OS either in high or low tumor mutation burden from Kaplan–Meier plotter databases.

The GEPIA database was used to evaluate the correlation of CD38 gene expression with the prognosis of ovarian cancer patients, and this analysis included 424 EOC cases. This analysis revealed that high levels of CD38 (above median) expression were associated with significantly longer disease-free survival (DFS, HR = 0.66, *P* = 0.00089) and increased OS (HR = 0.67, *P* = 0.0016) (Figures 2A,B).

To validate CD38 gene expression analysis, we next used the Kaplan–Meier plotter database to investigate the prognostic potential of CD38 expression in EOC, and this analysis included 1,657 patients with OS data and 1,435 patients with progression-free survival (PFS) data. CD38 gene expression was also strongly correlated with increased OS [HR = 0.75 (0.64–0.86), *P* = 0.0004] and PFS [HR = 0.8 (0.73–0.97), *P* = 0.0178] (Figures 2C,D and Table 1). The detailed relationships between CD38 mRNA expression and prognosis of EOC based on different clinicopathological characteristics in the Kaplan–Meier plotter database are presented in Table 1.

**TABLE 1:** Correlation of CD38 mRNA expression and clinical prognosis in ovarian cancer with different clinicopathological factors by Kaplan–Meier plotter.

	OS			PFS		
		
Clinicopathological traits	n	HR	*P*	n	HR	*P*
Total	1,656	0.75(0.64−−0.86)	1E-04	1,435	0.84(0.73−−0.97)	0.0178
Average CA-125 below lower quartile	395	0.6(0.46−−0.78)	0.00012	326	0.5(0.38−−0.66)	8.5E-07
**HISTOLOGY**						
Endometrioid	37	5.31(0.88−−31.88)	0.041	51	2.53(1.0−−6.45)	0.0431
Serous	1,207	0.7(0.6−−0.82)	1.3E-05	1,104	0.87(0.76−−1.01)	0.0639
**STAGE**						
I	74	1.74(0.52−−5.87)	0.3639	96	5.88(1.6−−21.64)	0.0028
II	61	1.91(0.62−−5.84)	0.2513	67	1.72(0.85−−3.49)	0.1287
III	1,044	0.69(0.59−−0.82)	2.2E-5	919	0.83(0.71−−0.97)	0.02
IV	176	0.67(0.45−−1.0)	0.0488	162	1.37(0.9−−2.07)	0.1357
**GRADE**						
I	56	1.62(0.61−−4.31)	0.3328	37	3.29(1.1−−9.83)	0.0236
II	324	0.69(0.49−−0.97)	0.0295	256	1.3(0.93−−1.82)	0.1211
III	1,015	0.65(0.55−−0.77)	8.7E-07	837	0.83(0.7−−0.99)	0.0346
**P53**						
Mutated	506	0.7(0.55−−0.89)	0.0043	483	0.71(0.56−−0.89)	0.0025
Wild type	94	1.36(0.74−−2.48)	0.318	84	1.55(0.88−−2.72)	0.1223
**DEBULK**						
Optimal	801	0.67(0.52−−0.87)	0.0022	696	0.84(0.7−−1.02)	0.0719
Suboptimal	459	0.72(0.59−−0.88)	0.0011	459	0.67(0.54−−0.83)	0.0002
**CHEMOTHERAPY**						
Contains platin	1,409	0.75(0.65−−0.86)	4.8E-05	1,259	0.75(0.66−−0.86)	2.2E-05
Contains Taxol	793	0.62(0.49−−0.78)	5.8E-05	715	0.79(0.65−−0.95)	0.0126
Contains Avastin	50	0.55(0.21−−1.43)	0.2168	50	0.75(0.39−−1.45)	0.391

*Bold values indicate P < 0.05.*

In Kaplan–Meier plotter databases, except the microarray analysis of CD38 expression, RNA sequencing data were also acquired and used for online analysis of the prognostic value of CD38 in 373 patients of EOC with diverse tumor mutation statuses. We found that CD38 levels were positively correlated with OS in patients with both high and low mutation burden (*P* = 0.0044 and 0.0027, respectively; Figures 2E,F).

### The Correlation of CD38 With Immune and Stromal Scores in EOC

**FIGURE 3:**
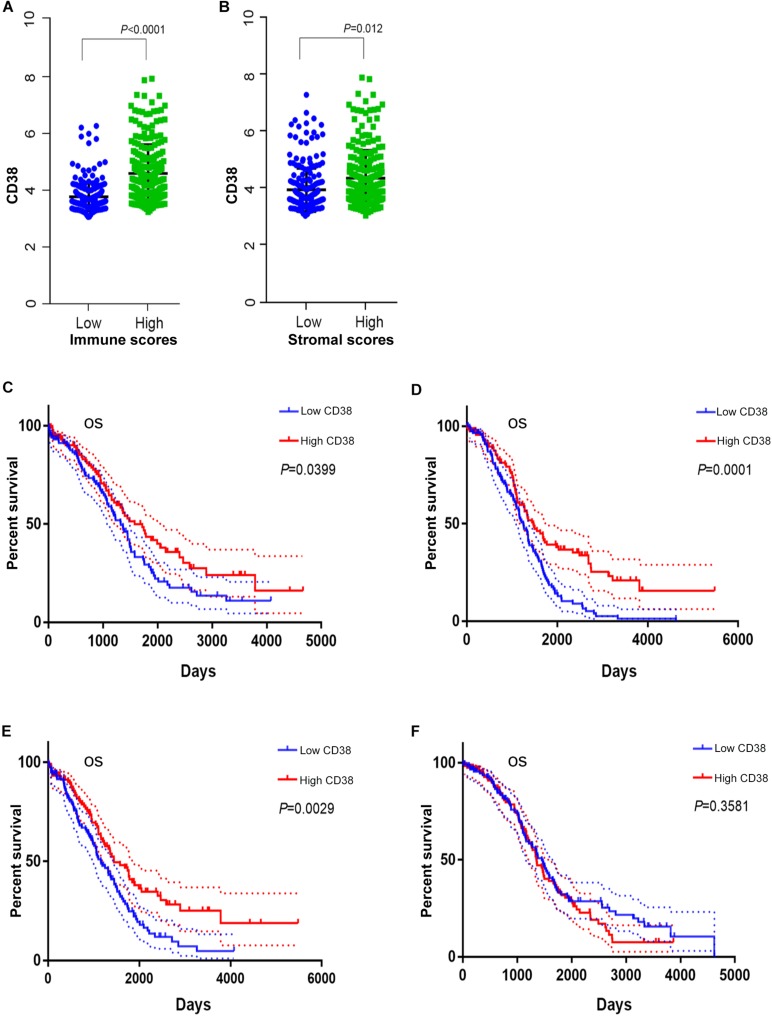
The correction between CD38 and immune or stromal scores (this analysis in ovarian cancer patients with immune or stromal scores median cutoff). **(A)** CD38 highly expressed in high immune scores group from TCGA database. **(B)** CD38 highly expressed in high stromal scores group from TCGA database. **(C)** Survival curves of OS in high immune scores group of epithelial ovarian cancer from TCGA database. **(D)** Survival curves of OS in low immune scores group of epithelial ovarian cancer from TCGA database. **(E)** Survival curves of OS in high stromal scores group of epithelial ovarian cancer from TCGA database. **(F)** Survival curves of OS in low stromal scores group of epithelial ovarian cancer from TCGA database.

The gene expression and clinical data profiles of 469 ovarian serous cystadenocarcinoma patients were downloaded from the TCGA database on May 10, 2019. The ESTIMATE algorithm was applied to assess stromal and immune cells in ovarian cancer. The analysis results implied that stromal scores of EOC were distributed from -1,988.05 to 1,837.43, and immune scores ranged from -1,498.58 to 2,774.16. To determine the potential relevance of CD38 with immune scores and/or stromal scores, 469 patients were classified into top (high group) and bottom halves (low group) according to their scores. Patients with high immune scores had higher CD38 expression compared with patients with low immune scores (Figure 3A). Consistently, patients with high stromal scores also showed higher CD38 expression compared with patients with low stromal scores (Figure 3B).

We further evaluated the prognostic impact of CD38 on the different statuses of immune scores and/or stromal scores for ovarian cancer. For the immune scores, CD38 gene expression was positively correlated with OS of EOC in both the high (above median) immune score group and the low score group (Figures 3C,D). The difference was that, for the stromal scores, CD38 gene expression was positively correlated with the OS of EOC in patients with high (above median) stromal scores but not in patients with low stromal scores (Figures 3E,F).

### CD38 Expression Is Involved in Antitumor Immunity

**FIGURE 4:**
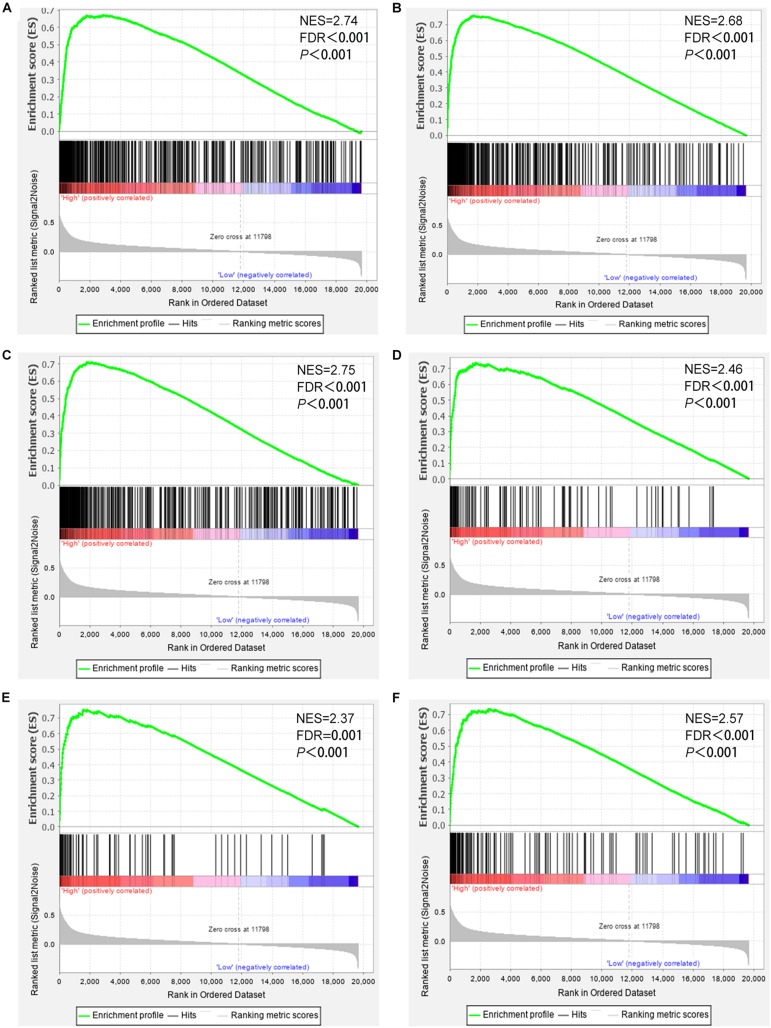
Gene set enrichment analysis showed that CD38 expression is involved in ovarian cancer patients’ antitumor immune responses. **(A)** Gene sets representing Innate immune response. **(B)** Adaptive immune response. **(C)** Lymphocyte activation. **(D)** Positive regulation of lymphocyte mediated immunity. **(E)** Regulation of T cell–mediated immunity. **(F)** Natural killer cell–mediated cytotoxicity.

To further study the roles of CD38 expression in the ovarian cancer microenvironment. Gene set enrichment analysis was conducted by utilizing the gene expression profiles of 469 EOC samples acquired from TCGA database, which contain RNA sequencing data. The gene signatures implied enrichment in many categories, such as immune response, adaptive immune response, lymphocyte activation, regulation of T cell–mediated immunity, and natural killer cell–mediated cytotoxicity, and were enriched in EOC samples with high CD38 expression (Figure 4). This analysis revealed that CD38 might play vital roles in antitumor immune modulation.

### The Relationship Between CD38 Expression and Immune Cell Infiltration

**FIGURE 5:**
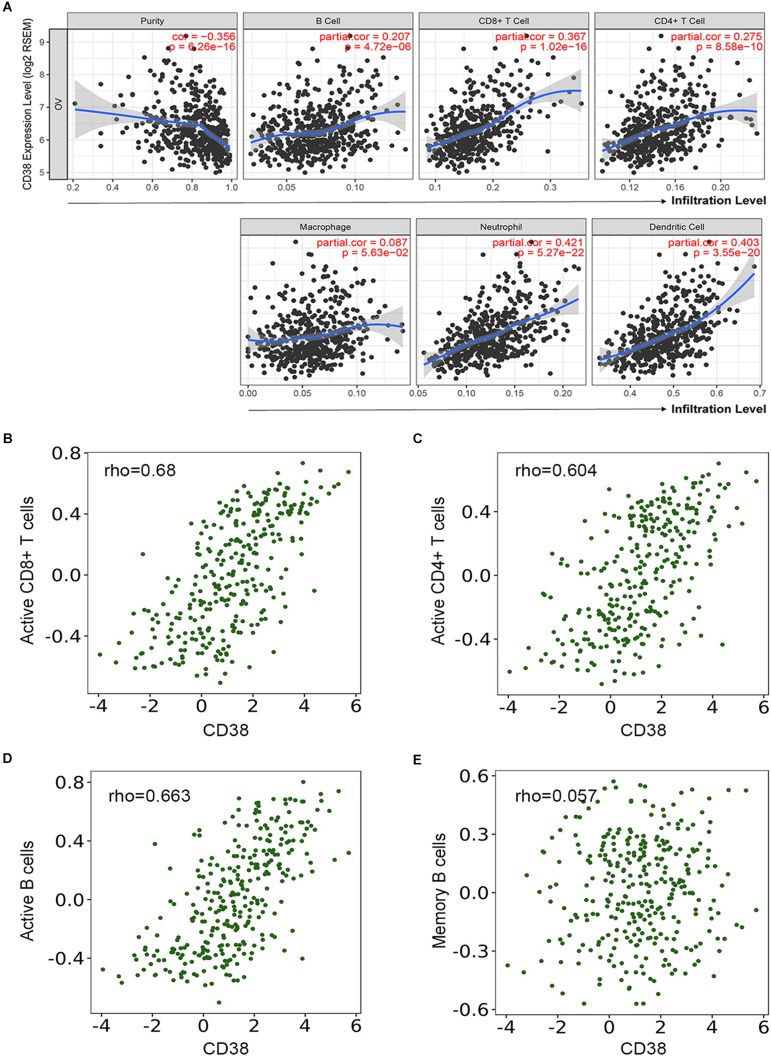
Correlation of CD38 expression with immune infiltration level in epithelial ovarian cancer. **(A)** CD38 expression is significantly negatively related to tumor purity and has significant positive correlations with infiltrating levels of B cells, CD8^+^ T cells, CD4^+^ T cells, macrophages, neutrophils, and dendritic cells from TIMER database. **(B–E)** CD38 expression has significant positive correlations with active CD8^+^ T cells, active CD4^+^ T cells, and active B cells, other than memory B cells.

Several studies have implied that TILs are a prognostic indicator for ovarian cancer (Zhang et al., 2003). Therefore, the associations between CD38 gene expression and TILs infiltration level in EOC were analyzed in the TIMER database. This analysis showed that CD38 was significantly correlated with tumor purity, CD8^+^ T cells, CD4^+^ T cells, and B cells in EOC. Myeloid cell types, including macrophages, neutrophils, and dendritic cells, were also significantly correlated with CD38 expression (Figure 5A). In the TISIDB database, we also found that CD38 was strongly related to immune infiltration in EOC, especially the infiltration of activated immune cells, such as activated CD8^+^ T cells (*R* = 0.68), activated CD4^+^ T cells (*R* = 0.604), and activated B cells (*R* = 0.663) (Figures 5B–D and Supplementary Table S2). Interestingly, the relationship between CD38 and memory immune cells was not strong (Figure 5E and Supplementary Table S3). To further clarify the relationship between CD38 and various subtypes of TILs in ovarian cancer, the TIMER and TISIDB online databases were employed to further analyze the relationship between CD38 and marker genes of different immune cells, including CD8^+^ T cells, CD4^+^ T cells, B cells, macrophages, neutrophils, and dendritic cells in EOC (Table 2 and Supplementary Table S3).

**TABLE 2:** Correlation analysis between CD38 and relate genes and markers of immune cells in TIMER.

		None		Purity	
			
Immune	Immune	Cor	*P*	Cor	*P*
profile	gene				
**T CELL**					
	CD3D	0.695	5.34E-45	0.654	1.03E-31
	CD3E	0.711	0.00	0.687319	3.71E-36
	CD3G	0.627	1.89E-34	0.543433	1.56E-20
	CD2	0.737	4.36E-53	0.718886	6.87E-41
**CD4^+^ T CELL**					
	CD4	0.535	0.00	0.468871	5.16E-15
**CD8^+^ T CELL**					
	CD8A	0.656	0.00	0.584927	2.98E-24
	CD8B	0.539	0.00	0.448	1.01E-13
	TBX21	0.738	2.51E-53	0.727225	2.99E-42
	EOMES	0.586	2.14E-29	0.512907	4.14E-18
	LCK	0.604	0.00	0.559081	7.10E-22
	IFNG	0.674	1.88E-41	0.610228	8.56E-27
	PRF1	0.687	0.00	0.663527	5.71E-33
	GZMA	0.655	0.00	0.629182	7.47E-29
	GZMB	0.665	5.01E-40	0.633114	2.68E-29
	GZMH	0.643	9.21E-37	0.59184	6.34E-25
	GZMK	0.625	3.56E-34	0.562898	3.26E-22
	GZMM	0.647	2.45E-37	0.615405	2.42E-27
	CXCL9	0.704	0.00	0.642913	1.95E-30
	CXCL10	0.792	0.00	0.767956	1.07E-49
**T_H_1**					
	IFNG	0.674	1.88E-41	0.610228	8.56E-27
	TBX21	0.738	2.51E-53	0.727225	2.99E-42
	TNF	0.247	1.46E-05	0.176	5.45E-03
	STAT4	0.673	2.98E-41	0.624	2.78E-28
	STAT1	0.641	0.00	0.61	1.02E-26
**T_H_2**					
	GATA3	0.277	1.04E-06	0.114	7.32E-02
	STAT6	0.066	2.55E-01	0.061	3.37E-01
	STAT5A	0.224	8.82E-05	0.216	5.84E-04
	IL13	0.167	3.48E-03	0.143	2.42E-02
**Tfh**					
	CXCR5	0.424	9.16E-20	0.338595	4.28E-08
	CXCL13	0.693	1.31E-44	0.618193	1.21E-27
	BCL6	–0.004	9.43E-01	0.067	2.93E-01
	IL21	0.325	7.11E-09	0.314	4.16E-07
**T_H_17**					
	IL17A	0.151	8.27E-03	0.118106	0.062768
	RORC	–0.114	4.82E-02	–0.04595	0.470446
	IL23A	0.076	1.88E-01	0.085397	0.179202
	STAT3	0.232	4.66E-05	0.153	1.56E-02
**Treg**					
	FOXP3	0.663	0.00	0.604827	3.12E-26
	IKZF2	–0.092	1.09E-01	–0.07343	0.248332
	IL10	0.321	1.31E-08	0.206341	0.001057
	TGFB1	0.362	1.09E-10	0.194492	0.002049
	CCR8	0.49	9.65E-20	0.408	2.03E-11
	STAT5B	–0.061	2.90E-01	–0.079	2.13E-01
**CHECKPOINTS**					
	CTLA4	0.738	2.14E-53	0.70801	3.46E-39
	PDCD1	0.609	3.67E-32	0.558032	8.78E-22
	LAG3	0.764	0.00	0.750288	2.73E-46
	PDL1/CD274	0.682	0.00	0.642702	2.07E-30
	TIM3/HAVCR2	0.578	0.00	0.512411	4.51E-18
	TIGIT	0.733	3.26E-52	0.694518	3.50E-37
**PROINFLAMMATION**					
	PTGS2	0.11	5.67E-02	–0.00507	0.936513
	IL8	0.081	1.59E-01	0.006259	0.921719
	IL1A	0.098	8.77E-02	0.02809	0.659131
	IL1B	0.305	7.07E-08	0.169372	0.007393
	IL18	0.32	1.48E-08	0.245112	9.30E-05
	IL6	0.273	1.36E-06	0.134232	0.034253
	IL12A	0.223	9.37E-05	0.179456	0.004503
	TNF	0.247	1.46E-05	0.175629	0.005451
**METABOLISM**					
	IDO1	0.584	0.00	0.489875	1.96E-16
	NOS2	–0.021	7.13E-01	–0.08555	0.178423
	HIF1A	0.01	8.63E-01	–0.07241	0.254943
**APC/DC**					
	HLA-DPA1	0.559	0.00	0.484698	4.48E-16
	HLA-DPB1	0.519	0.00	0.433577	7.79E-13
	HLA-DQA1	0.474	0.00	0.376692	8.15E-10
	HLA-DRA	0.508	0.00	0.431049	1.09E-12
	HLA-DMA	0.456	0.00	0.392215	1.39E-10
	HLA-DQB1	0.36	1.36E-10	0.275	1.05E-05
	BDCA-1/CD1C	0.191	8.09E04	0.073	2.53E-01
	BDCA-4/NRP1	0.176	2.18E-03	0.038	5.54E-01
	CD11C/ITGAX	0.489	0.00	0.422	3.41E-12
**B CELL**					
	BLK	0.325	7.28E-09	0.245509	9.05E-05
	CD19	0.352	2.85E-10	0.3409	3.42E-08
	MS4A1	0.57	01.75E-27	0.478648	1.16E-15
	CD79A	0.62	1.43E-33	0.522	8.55E-19
**MONOCYTE**					
	CD86	0.639	0.00	0.579	1.02E-23
	CD115/CSF1R	0.411	7.85E-14	0.306	8.61E-07
**TAM**					
	CCL2	0.428	0.00	0.359	5.38E-09
	CD68	0.584	0.00	0.532	1.33E-19
	CSF2	0.338	1.56E-09	0.318	3.03E-07
**M1**					
	INOS/NOS2	–0.021	7.13E-01	–0.086	1.78E-01
	IRF5	0.263	3.65E-06	0.236	1.75E-04
	COX2/PTGS2	0.11	5.67E-02	–0.005	9.37E-01
**M2**					
	CD163	0.511	0.00	0.424	2.70E-12
	VSIG4	0.438	0.00	0.33	9.62E-08
	MS4A4A	0.539	0.00	0.484	4.73E-16
**N**					
	CD66B/CEACAM8	–0.094	1.01E-01	–0.083	1.93E-01
	CD11B/ITGAM	0.454	0.00	0.373	1.25E-09
	CCR7	0.65	0.00	0.614	3.20E-27
**NK**					
	KIR2DL1	0.225	7.82E-05	0.135	3.37E-02
	KIR2DL3	0.24	2.46E-05	0.212	7.38E-04
	KIR2DL4	0.53	2.67E-23	0.497	6.38E-17
	KIR3DL1	0.392	1.44E-12	0.353	1.03E-08
	KIR3DL2	0.188	9.88E-04	0.134	3.40E-02
	KIR3DL3	0.148	9.94E-03	0.12	5.96E-02
	KIR2DS4	0.19	9.16E-04	0.128	4.32E-02

Cor, R value of Spearman correlation; None, correlation without adjustment; Purity, correlation adjusted by purity. *P < 0.01; **P < 0.001; ***P < 0.0001.

## Discussion

As a multifunctional ADP-ribosyl cyclase, CD38 is widely expressed on plasma cells and other types of immune cells (Deaglio et al., 2001). With daratumumab (an anti-CD38 mAb) approved for clinical application, CD38 has emerged as a high-impact therapeutic target in multiple myeloma (Nijhof et al., 2015; Elsada and Adler, 2019). The CD38/CD203a/CD73 adenosinergic pathway is a major regulatory mechanism in niche metabolic reprogramming (Horenstein et al., 2013). Furthermore, CD38 is expressed on various lymphocytes, including regulatory T cells (Tregs), B cells, and myeloid cells, which have potential immunomodulatory effects (Flores-Borja et al., 2013; Karakasheva et al., 2015; Feng et al., 2017). However, the role of immunologic reprogramming in the solid TME is still unclear. Here, we present a study that revealed that CD38 expression levels correlate with prognosis in ovarian cancer. High expression of CD38 correlates with early disease stage and better prognosis. In addition, our analyses show that TILs and diverse immune markers in ovarian cancer are associated with CD38 expression levels. Hence, our comprehensive and systematic analysis study provides valuable insights into the potential immune regulatory role of CD38 in the EOC niche and suggests its use as a cancer prognostic biomarker.

Our study analyzed the CD38 mRNA expression level in normal ovaries and EOC by using online datasets in GEPIA, Oncomine, and TISIDB. The expression of the CD38 gene in EOC was not only higher than that in normal tissue but was also higher than that in borderline ovarian tumors. Nevertheless, ovarian cancer is not a single disease and can be subdivided into many molecular subtypes. Analysis of the TISIDB database showed that the CD38 gene had the highest expression level in the immunoreactive subtype, followed by the mesenchymal type, with little expression in the differentiated and proliferative types. Different levels of CD38 expression in distinct immune subtypes of ovarian cancer were observed, and the C2 (IFN-γ dominant) type had the highest level compared with the other three subtypes. The comprehensive and detailed analysis of CD38 gene expression in various databases among EOC and different subtypes may reflect that CD38 is strongly linked to immunological properties in the microenvironment.

Nevertheless, in the Kaplan–Meier plotter and GEPIA databases, the analysis found matching prognostic value correlations between CD38 expressions in EOC. The increased CD38 expression correlated with better survival in EOC and was not influenced by the immune scores. In addition, high CD38 expression was related to favorable prognosis of EOC in stages III and IV and grades II and III. Together, these results robustly indicated that CD38 is a potential prognostic biomarker for ovarian cancer.

Another important finding is that CD38 expression is closely related to the immune response and lymphocyte infiltration in EOC. Under physiological conditions, CD38 induced mature B-cell proliferation and immunoglobulin M (IgM) secretion. And in CD38 expressed higher on activated T cells, the CD38^+^ T cells inhibited CD38^–^ T-cell proliferation to maintain T-cell homeostasis (Bahri et al., 2012; Glaria and Valledor, 2020). On the contrary, another study have unveiled that T cells expressing high levels of CD38 have an extremely low proliferative ability but an enhanced capacity to produce interleukin 2 (IL-2) and IFN-γ (Sandoval-Montes and Santos-Argumedo, 2005). These evidences all suggested that CD38 plays a vital role in the regulation of immune cells activation and differentiation. But its exact regulatory function still needs further study. The GSEA and correlation analyses in our study implied that CD38 regulated the tumor immune microenvironment in EOC and was associated with B- and T-cell activation and regulated immune responses. A study also certified that in human lung cancer CD38 protein is highly expressed in CD8^+^ tissue-resident memory cells, CD103^+^ (T_RM_ cells), and a high density of T_RM_ cell infiltration predicts a better prognosis (Ganesan et al., 2017).

Another study revealed that CD38 is one of the essential mechanisms by which tumors obtain resistance to immune checkpoint blockade immunotherapy, resulting in CD8^+^ T-cell dysfunction. Interferon β might be a factor increasing CD38 expression in the TME (Chen et al., 2018). In addition, Schietinger et al. certified that PD1^hi^ TILs were a heterogeneous population and that PD1^hi^ T cells with increased CD38 expression did not respond to PD-1 and/or PD-L1 immune checkpoint blockers. CD38^+^ PD1^hi^ T cells may be in a fixed dysfunctional state rather than the plastic reprogrammable state (Philip et al., 2017). All of the studies hinted that CD38 plays a vital role in remodeling the immune microenvironment, and CD38 deserves further research as an immunotherapeutic target and prognostic biomarker in ovarian cancer.

## Data Availability Statement

The datasets analyzed for this study can be found in the GEPIA (http://gepia.cancer-pku.cn/index.html), Oncomine (http://www.oncomine.org), TISIDB (http://cis.hku.hk/TISIDB), Tumor Immune Estimation Resource (TIMER, https://cistrome.shinyapps.io/timer), TCGA databases (https://tcga-data.nci.nih.gov/tcga/).

## Author Contributions

JZ and ZZ: study concept and design. YZ, ZJ, and YL: acquisition and analysis of the data. JZ, YZ, and ZZ: drafting and revising of the manuscript.

## Conflict of Interest

The authors declare that the research was conducted in the absence of any commercial or financial relationships that could be construed as a potential conflict of interest. The reviewer XC and handling Editor declared their shared affiliation.

## Footnotes

1 http://gepia.cancer-pku.cn/index.html

2 http://www.oncomine.org

3 http://cis.hku.hk/TISIDB

4 www.kmplot.com

5 https://cistrome.shinyapps.io/timer

6 https://tcga-data.nci.nih.gov/tcga/

7 http://www.broadinstitute.org/gsea/

## Supplementary Material

FIGURE S1 CD38 expression levels in different types of epithelial ovarian tumor. **(A)** CD38 in data sets of epithelial ovarian cancer compared with borderline ovarian tumor in the Oncomine database. **(B)** CD38 in data sets of ovarian serous cancer compared with ovarian endometrioid cancer in the Oncomine database.

TABLE S1 Detailed information of the online databases applied in the study.

TABLE S2 Spearman correlation analysis between expression of CD38 and TILs in epithelial ovarian cancer from TISIDB database.

TABLE S3 Spearman correlation analysis between expression of CD38 and Immunomodulator in epithelial ovarian cancer from TISIDB database.
