# Supplementary figures and images for: CD38 Predicts Favorable Prognosis by Enhancing Immune Infiltration and Antitumor Immunity in the Epithelial Ovarian Cancer Microenvironment
Source: Front Genet. 2020 Apr 30;11:369. doi: 10.3389/fgene.2020.00369 (PMC7203480; doi:10.3389/fgene.2020.00369)

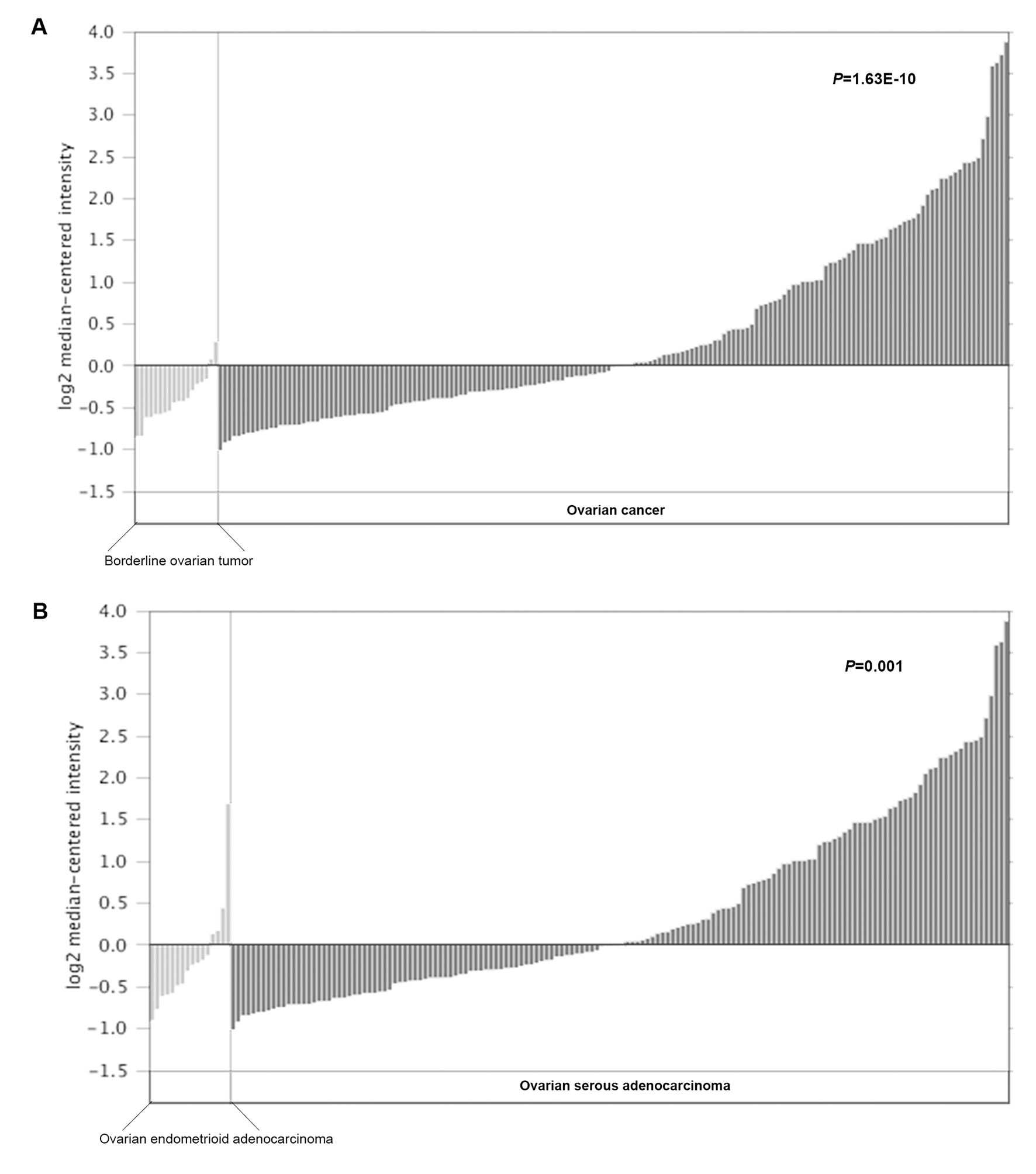

Supplement: FIGURE S1 — CD38 expression levels in different types of epithelial ovarian tumor. (A) CD38 in data sets of epithelial ovarian cancer compared with borderline ovarian tumor in the Oncomine database. (B) CD38 in data sets of ovarian serous cancer compared with ovarian endometrioid cancer in the Oncomine database. [file Image_1.TIF]
