# Supplementary material for: CD38 Predicts Favorable Prognosis by Enhancing Immune Infiltration and Antitumor Immunity in the Epithelial Ovarian Cancer Microenvironment
Source: Front Genet. 2020 Apr 30;11:369. doi: 10.3389/fgene.2020.00369 (PMC7203480; doi:10.3389/fgene.2020.00369)
Supplement: TABLE S1 — Detailed information of the online databases applied in the study. [file Table_1.DOCX]

**Supplementary Table 1: Detailed information of the online databases applied in the study.**

| **Databases** | **IDs/sources** | **Ovarian cancer**  **histological type** | **Samples** | | **Applied in the study** | | |
| --- | --- | --- | --- | --- | --- | --- | --- |
|  |  |  | **Tumor** | **Normal** | **mRNA expression analysis** | **Prognostic value analysis** | **Immune correction analysis** |
| GEPIA Database | http://gepia.cancer-pku.cn | Serous ovarian cancer | 426 | 88 | √ | √ |  |
| TISIDB Database | http://cis.hku.hk/TISIDB | Serous ovarian cancer | NA | 0 |  |  | √ |
| Kaplan-Meier Database | http://kmplot.com | Serous and endometrioid ovarian cancer | 1232 | 0 |  | √ |  |
| TIMER Database | https://cistrome.shinyapps.io/timer | Serous ovarian cancer | 303 | 0 |  |  | √ |
| NA: Not Available. The genomics, transcriptomics and clinical data of TISIDB are from The Cancer Genome Atlas (TCGA) database | | | | | | | |
