# Supplementary material for: CD38 Predicts Favorable Prognosis by Enhancing Immune Infiltration and Antitumor Immunity in the Epithelial Ovarian Cancer Microenvironment
Source: Front Genet. 2020 Apr 30;11:369. doi: 10.3389/fgene.2020.00369 (PMC7203480; doi:10.3389/fgene.2020.00369)
Supplement: TABLE S2 — Spearman correlation analysis between expression of CD38 and TILs in epithelial ovarian cancer from TISIDB database. [file Table_2.DOCX]

**Supplementary Table 2: Spearman correlation analysis between expression of CD38 and TILs in epithelial ovarian cancer from TISIDB database.**

| **Tumor-infiltrating lymphocytes** | **R value** | ***P* value** |
| --- | --- | --- |
| Activated CD8+ T cells | 0.68 | 0 |
| Central memory CD8+ T cells | 0.351 | 3.14E-10 |
| Effector memory CD8+ T cells | 0.609 | 0 |
| Activated CD4+ T cells | 0.604 | 0 |
| Central memory CD4+ T cells | 0.322 | 9.86E-09 |
| Effector memory CD4+ T cells | 0.37 | 2.97E-11 |
| T follicular helper cells | 0.495 | 0 |
| γδT cells | 0.296 | 1.43E-07 |
| Type 1 T helper cells | 0.533 | 0 |
| Type 17 T helper cells | 0.337 | 1.64E-09 |
| Type 2 T helper cells | 0.25 | 1E-05 |
| Regulatory T cells | 0.511 | 0 |
| Activated B cells | 0.663 | 0 |
| Immature B cells | 0.712 | 0 |
| Memory B cells | 0.057 | 0.32 |
| Natural killer cells | 0.405 | 1.66E-13 |
| CD56bright natural killer cells | 0.316 | 1.8E-08 |
| CD56dim natural killer cells | 0.251 | 9.12E-06 |
| Myeloid derived suppressor cells | 0.594 | 0 |
| Natural killer T cells | 0.509 | 0 |
| Activated dendritic cells | 0.432 | 0 |
| Plasmacytoid dendritic cells | 0.269 | 1.96E-06 |
| Immature dendritic cells | 0.211 | 0.000198 |
| Macrophage | 0.437 | 0 |
| Eosinophil | 0.3 | 9.73E-08 |
| Mast cells | 0.468 | 0 |
| Monocyte | 0.33 | 3.71E-09 |
| Neutrophil | 0.299 | 1.07E-07 |
| **P* < *0.01;* ***P* < *0.001;* ****P* < *0.0001.* | | |
