# Supplementary material for: CD38 Predicts Favorable Prognosis by Enhancing Immune Infiltration and Antitumor Immunity in the Epithelial Ovarian Cancer Microenvironment
Source: Front Genet. 2020 Apr 30;11:369. doi: 10.3389/fgene.2020.00369 (PMC7203480; doi:10.3389/fgene.2020.00369)
Supplement: TABLE S3 — Spearman correlation analysis between expression of CD38 and Immunomodulator in epithelial ovarian cancer from TISIDB database. [file Table_3.DOCX]

**Supplementary Table 3: Spearman correlation analysis between expression of CD38 and** [**Immunomodulator**](http://cis.hku.hk/TISIDB/browse.php?gene=CD38#inhibitor) **in epithelial ovarian cancer from TISIDB database.**

| [**Immunomodulator**](http://cis.hku.hk/TISIDB/browse.php?gene=CD38#inhibitor) | **R value** | ***P* value** |
| --- | --- | --- |
| [**Immunoinhibitor**](http://cis.hku.hk/TISIDB/browse.php?gene=CD38#inhibitor) | | |
| ADORA2A | 0.474 | 0 |
| BTLA | 0.298 | 1.23E-07 |
| CD160 | 0.155 | 0.00644 |
| CD244 | 0.583 | 0 |
| CD274 | 0.687 | 0 |
| CD96 | 0.661 | 0 |
| CSF1R | 0.4 | 3.99E-13 |
| CTLA4 | 0.734 | 0 |
| HAVCR2 | 0.573 | 0 |
| IDO1 | 0.538 | 0 |
| IL10 | 0.306 | 5.07E-08 |
| IL10RB | 0.073 | 0.2 |
| KDR | 0.039 | 0.493 |
| LAG3 | 0.767 | 0 |
| LGALS9 | 0.488 | 0 |
| PDCD1 | 0.609 | 0 |
| PDCD1LG2 | 0.719 | 0 |
| PVRL2 | -0.091 | 0.111 |
| TGFB1 | 0.349 | 4.39E-10 |
| TGFBR1 | -0.1 | 0.0802 |
| TIGIT | 0.733 | 0 |
| VTCN1 | 0.299 | 1.06E-07 |
| [**Immunostimulator**](http://cis.hku.hk/TISIDB/browse.php?gene=CD38#stimulator) | | |
| CD27 | 0.705 | 0 |
| CD276 | -0.088 | 0.124 |
| CD28 | 0.379 | 8.42E-12 |
| CD40 | 0.375 | 1.56E-11 |
| CD40LG | 0.655 | 0 |
| CD48 | 0.683 | 0 |
| CD70 | 0.469 | 0 |
| CD80 | 0.68 | 0 |
| CD86 | 0.634 | 0 |
| CXCL12 | 0.175 | 0.00217 |
| CXCR4 | 0.161 | 0.00462 |
| ENTPD1 | 0.22 | 0.000109 |
| HHLA2 | 0.067 | 0.239 |
| ICOS | 0.788 | 0 |
| ICOSLG | 0.026 | 0.652 |
| IL2RA | 0.576 | 0 |
| IL6 | 0.259 | 4.66E-06 |
| IL6R | 0.303 | 7.15E-08 |
| KLRC1 | 0.448 | 0 |
| KLRK1 | 0.657 | 0 |
| LTA | 0.685 | 0 |
| MICB | 0.386 | 3.48E-12 |
| NT5E | 0.134 | 0.0189 |
| PVR | -0.002 | 0.978 |
| TMEM173 | 0.022 | 0.697 |
| TMIGD2 | 0.515 | 0 |
| TNFRSF13C | 0.099 | 0.0848 |
| TNFRSF14 | 0.313 | 2.63E-08 |
| TNFRSF17 | 0.589 | 0 |
| TNFRSF18 | 0.35 | 3.78E-10 |
| TNFRSF25 | 0.04 | 0.481 |
| TNFRSF4 | 0.428 | 0 |
| TNFRSF8 | 0.244 | 1.58E-05 |
| TNFRSF9 | 0.471 | 0 |
| TNFSF13 | 0.205 | 0.000299 |
| TNFSF13B | 0.73 | 0 |
| TNFSF14 | 0.287 | 3.43E-07 |
| TNFSF15 | 0.184 | 0.00121 |
| TNFSF4 | 0.134 | 0.0189 |
| TNFSF9 | 0.167 | 0.0033 |
| ULBP1 | 0.033 | 0.565 |
| **P* < *0.01;* ***P* < *0.001;* ****P* < *0.0001.* | | |
